# 

**Published:** 1877-10

**Authors:** 


### CONTENTS.

### COMMUNICATIONS.

The Unity of Force, Electricity, its Superlative Velocity—*J. J. Caldwell, M. D*.... 405

What is Dentistry—The Dignity of the Profession—The Standard to which it should be Raised—*Dr. J. D. Miles*.... 410

Tennessee Dental Association.... 414

The Development of Dentine—*Chr. Sihler, M. D* .... 423

Proceedings of the Mississippi State Dental Association.... 437

A Case of Suppression of Salivary Secretion—*Dr. Donald Baynes* .... 441

### SELECTIONS.

A Good Cheap Paste .... 443

Salt for the Throat.... 443

Nicotinism.... 444

Tincture Gelseminum.... 444

### EDITORIAL.

Annual Meeting Connecticut Valley Dental Association.... 445

Text Books.... 445

The Physician's Visiting List .... 445

Educational.... 446

Use of Tin Foil by Junior Practitioners.... 447

